# Advances in Nanolime and Other Nanomaterials for Built Heritage Conservation

**DOI:** 10.3390/ma16041565

**Published:** 2023-02-13

**Authors:** Luis Monasterio-Guillot, Giovanni Borsoi, Jorge Otero

**Affiliations:** 1 Department of Mineralogy and Petrology, University of Granada, 18071 Granada, Spain; 2 ISTerre, Université Grenoble Alpes, 38000 Grenoble, France; 3 Civil Engineering Research and Innovation for Sustainability (CERIS), Technical University of Lisbon, 1049-001 Lisbon, Portugal

This Special Issue examines the synthesis, characterization, and manufacturing of nanoparticles and their potential advantages and applications for the conservation of built cultural heritage materials.

Machu Picchu, Petra, Angkor, Copán, Taj Mahal, and the Easter Island Moai are among many other monuments with irreplaceable cultural heritage significance, and all are currently eroding at a noticeable rate. This is leading to a constant loss of these structures’ cultural, artistic, and economic value. This rapid deterioration is being exacerbated by our lack of understanding of adequate remedial strategies to preserve these historical sites. Many attempts are currently being made by scientists worldwide toward the improvement of conservation actions, focusing mostly on the cleaning and consolidation of these substrates [1,2]. Within this context, innovative methods, including the use of novel nanomaterial products, are being investigated as consolidant agents and/or protective coatings that could induce structural self-cleaning, insulation, air-purifying, or solar protection properties [3,4].

This Special Issue welcomes contributions that address research and practical advances in: (i) the novel synthesis and manufacturing process of nanoparticles; (ii) chemical, microstructural, and structural characterization of nanoparticles; (iii) evaluation of the cleaning or consolidation effectiveness; (iv) product application methods; (v) assessments of the long-term characteristics and durability of treated structures; and (vi) remarkable case studies.

This Special Issue aims to answer some of those questions while presenting many considerable recent advances in nanolimes and other nanomaterials, as well as their applications for the conservation of our built heritage. All articles in this Special Issue will focus on papers with a broad interest to the built heritage community and their conservation practitioners, especially novel, high-quality studies with potential for real-world applicability. 
